# A Magneto-Viscoelasticity Problem with Aging

**DOI:** 10.3390/ma15217810

**Published:** 2022-11-05

**Authors:** Sandra Carillo, Claudio Giorgi

**Affiliations:** 1 Dipartimento Scienze di Base e Applicate per l’Ingegneria, SAPIENZA Università di Roma, 00161 Roma, Italy; 2 I.N.F.N.-Sez. Roma1, 00185 Roma, Italy; 3 Dipartimento di Ingegneria Civile, Architettura, Territorio, Ambiente e di Matematica, Università di Brescia, 25133 Brescia, Italy

**Keywords:** magneto-mechanic interactions, magneto-viscoelasticity, aging materials with memory, integro-differential evolution equation

## Abstract

This study addresses a magneto-viscoelasticity problem, considering the one-dimensional case. The system under investigation is given by the coupling a non-linear partial differential equation with a linear integro-differential equation. The system models a viscoelastic body whose mechanical behavior, described by the linear integro-differential equation, is also influenced by an external magnetic field. The model here investigated aims to consider the concomitance of three different effects: viscoelasticity, aging and magnetization. In particular, the viscoelastic behavior is represented via an integro-differential equation whose kernel characterizes the properties of the material. In a viscoelastic material subject to the effects of aging, all changes in the response to deformation are due not only to the intrinsic memory of the material but also to deterioration with the age of the material itself. Thus, the relaxation function is not assumed to depend on the two times, present and past, via their difference, but to depend on both the present and past times as two independent variables. The sensibility to an external magnetic field is modeled by a non-linear partial differential equation taking its origin in the Landau–Lifschitz magnetic model. This investigation is part of a long-term research project aiming to provide new insight in the study of materials with memory and, in particular, viscoelastic materials. Specifically, the classical model of viscoelastic body introduced by Boltzmann represents the fundamental base from which a variety of generalizations have been considered in the literature. In particular, the effects on the viscoelastic body due to interaction with an external magnetic field are studied. The new aspect under investigation is the combined presence of the external magnetic field with the effect of aging. Indeed, the coupling of viscoelasticity, which takes into account the deterioration of the material with time, with the presence of an external magnetic field, was never considered in previous research. An existence and uniqueness result is proved under suitable regularity assumptions.

## 1. Introduction

The study of magneto-viscoelastic materials finds its motivation in a wide variety of new materials. Indeed, the possibilIty of constructing a viscoelastic material which is characterized by mechanical response which can be modified under the action of an external magnetic field turned out to be of importance in different applications [1,2,3]. As an example, magneto-viscoelastic materials are considered in biomedical and seismic applications. The key fact in biomedicine is that the most appropriate model of human tissues, when the mechanical properties are concerned, is that of a suitable viscoelastic media. Notable examples are represented by human bones which, in most cases, cannot be modeled as solids nor as fluids, and thus, the viscoelasticity model seems more appropriate. The idea to adopt the viscoelasticity model in the case of human bones was already present in the literature in 1976 [4] and was subsequently developed in various directions, such as the possible changes of the viscoelastic properties of human bones due to pathological reasons. Under this viewpoint in [5], the changes in the viscoelastic response are investigated in the case of patients affected by diabetes, and also, aging effects are mentioned. As far as viscoelastic models of bones are concerned, in [6] the viscoelastic damage is considered subject to magnetization and electric polarization. In addition, thermal sensibility is also expected and, hence, a thermo-magneto-viscoelasticity model studied under an experimental viewpoint deserves a mathematical investigation.

In other cases, more closely related to the present investigation, the viscoelastic properties of bones are investigated aiming to devise artificial implantations. Indeed, osteoporosis effects are among the aging effects. A further example of viscoelastic material widely studied is the brain. According to the review [7], which deals with how the viscoelasticity model can be used in this field, investigations aim to provide the most appropriate model for modeling traumatic injuries. The interest in magneto-sensible viscoelastic materials can be found also in biomedical applications [8], where the use of magneto-sensible hydrogels is suggested in drug delivery [9]. Indeed, the presence of micro or nanoparticles within a viscoelastic material gives the opportunity to influence the mechanical behavior of the material itself when an external magnetic field is applied or tuned.

Viscoelastic materials which exhibit a magnetic sensibility find interesting use also in seismic applications [10]. Magneto-rheological elastomers are devised to prevent, as much as possible, damages to buildings in case of earthquakes. In this case, according to [1,11], unbounded relaxation functions are appropriate [12]. Finally, it can be mentioned that magneto-active polymers are also of interest in aerospace applications [13].

The present investigation is part of a long-term research project devoted to studying the analytical properties of systems which model mechanical properties of materials with memory. In [14,15,16], magneto-elasticity problems are analyzed, whereas in [17,18], problems arising in magneto-viscoelasticity are studied. In these papers, the cases of one-dimensional as well as three-dimensional bodies are studied, assuming that the kernel representing the relaxation modulus is regular or singular.

The novelty of our contribution consists of taking into account also the aging of the material. Specifically, magneto-viscoelastic solids subject to aging under the assumption of a constant temperature are investigated. Accordingly, the model of aging isothermal viscoelasticity is adopted.

Specifically, for the reader’s convenience, the results are organized as follows. Section 2 is devoted to the introduction of the model of viscoelastic body with aging, which is how the relaxation modulus is modified to take into account that the mechanical response of the material changes over time due to modifications within the viscoelastic body itself. Indeed, in the case under investigation, the kernel in the integral term, which represents the relaxation modulus, depends on both the present as well as the past time, which are regarded as two independent variables. Notably, the classical viscoelasticity model is obtained when the relaxation modulus depends on time via the difference between the two times, i.e., present and past times.

In Section 3, the one-dimensional magneto-viscoelasticity problem is introduced. In particular, the two effects, viscoelasticity and magneto sensibility are synthetized in a system of two equations, respectively, a linear integro-differential and a non-linear partial differential equation. The same section provides also the a priori estimates crucial to demonstrate the existence and uniqueness result aimed for. Remarkably, a key estimate is obtained only in terms of the viscoelasticity term. A further two estimates refer to the interaction between the two different physical phenomena. In the following Section 4, the main result is presented. Specifically, in Section 4, under suitable regularity conditions, the proof of the existence and uniqueness of weak solution is constructed, firstly locally in time; then, it is extended.

## 2. Aging Viscoelastic Body

The viscoelastic body is assumed to be homogeneous, which implies that the dependence on the spatial variable can be omitted in the description of its behavior. On the converse, the dependence on time is assumed to be not only through the present time but also on the past (deformation) history of the material. The environment which surrounds the body is understood to be passive, i.e., it is not influenced by the presence, or the status, of the viscoelastic body.

The mechanical status of the viscoelastic body is described on imposing constitutive assumptions whose aim is to guarantee the physical meaningfulness of the model (see, for instance, [19]). In classical linear viscoelasticity, the quantities involved are: the strain tensor, E=E(t), defined as $E=\frac{1}{2}\left(\nabla u+\nabla u^{T}\right)$, and the Cauchy stress tensor T=T(t). At any point of the body, the stress at any time *t* depends upon the strain at all preceding times τ<t.

According to the pioneering work of Boltzmann, the stress–strain relation was taken to be linear so that a superposition of the influence of previous strains hold. In addition, the influence of a previous strain on the stress depends on the time elapsed since that strain occurred and is weaker for those strains that occurred long ago. After introducing the elapsed time, s:=t−τ, memory weakening is governed by the relaxation modulus G=G(s), $s\in {I\;R}^{+}$, which assume the initial value denoted as $G_{0}:=G\left(0\right)$. For any given t∈IR, the linear stress–strain relation is given by

(1) $$T\;\left(t\right)=G_{0}E\left(t\right)+\int_{-\infty }^{t}G^{\prime }\left(t-\tau \right)E\left(\tau \right)d\tau \;\;,\;\;G\left(s\right)=G_{0}+\int_{0}^{s}G^{\prime }\left(\xi \right)\;d\xi .$$

where a prime mark denotes the derivation of the function with respect to its argument. After introducing the strain past history, $E^{t}\left(s\right):=E\left(t-s\right)$, and making a change of variables, this relation takes the form

(2) $$T\;\left(t\right)=G_{0}E\left(t\right)+\int_{0}^{\infty }G^{\prime }\left(s\right)E^{t}\left(s\right)ds.$$

In addition, G enjoys fading memory property, that is

**Proposition** **1.** *For all ε>0, there exists $\tilde{a}=a\;\left(\varepsilon ,E^{t}\right)\in {I\;R}^{+}$ such that*

(3) $$\left|\int_{0}^{\infty }\;\;G^{\prime }\left(s+a\right)E^{t}\left(s\right)\;ds\right|\;<\;\varepsilon \;,\;\forall a>\tilde{a}.$$

The classical assumptions the relaxation modulus satisfies are:

(4) $$G^{\prime }\in L^{1}\left({I\;R}^{+}\right)\;\;,\;\;G\left(t\right)=G_{0}+\int_{0}^{t}G^{\prime }\left(s\right)\;ds\;,\;G_{\infty }=\lim_{t\to \infty }G\left(t\right).$$

The relaxation modulus $G_{\infty }$ is positive definite in solids.

When aging effects are modeled, it can be assumed that the dependence of the relaxation modulus G on *t* and τ is not only through their difference t−τ as in (1) but involves *t* and τ separately, namely G(t,τ) (see, for instance, [19,20,21]). The classical expression is recovered by simply assuming that

G(t,τ)=G(t−τ),τ≤t.

In particular,

$$G_{\tau }\left(t,\tau \right)=-G^{\prime }\left(t-\tau \right),\;G\left(t,t\right)=G_{0},$$

where in the subscript ${}_{\tau }$ indicates partial derivative with respect to τ. Aging effects are taken into account by modifying the stress–strain relation (2) as follows

(5) $$T\;\left(t\right)=G\left(t,t\right)E\left(t\right)-\int_{-\infty }^{t}G_{\tau }\left(t,\tau \right)E\left(\tau \right)d\tau .$$

In view of further applications, it can be convenient to introduce the tensor-valued function G, defined on $I\;R\times {I\;R}^{+}$ as

G(t,s)=G(t,t−s),

and hence

$$G_{s}\left(t,s\right)=G_{s}\left(t,t-s\right)=-G_{\tau }\left(t,\tau \right),\;\tau =t-s.$$

This function depends not only on the elapsed time *s* but also on the current time *t*, thus representing the effects of aging. Accordingly, (5) becomes

(6) $$T\;\left(t\right)=G_{0}\left(t\right)E\left(t\right)+\int_{0}^{\infty }G_{s}\left(t,s\right)E^{t}\left(s\right)ds.$$

where $G_{0}\left(t\right):=G\left(t,0\right)=G\left(t,t\right)$, and

$$G\left(t,s\right)=G_{0}\left(t\right)+\int_{0}^{s}G_{\xi }\left(t,\xi \right)\;d\xi ,\;G_{\infty }\left(t\right)=\lim_{s\to +\infty }G\left(t,s\right).$$

In addition, the reduced kernel

$$\overset{ˇ}{G}\left(t,s\right)=G\left(t,s\right)-G_{\infty }\left(t\right)$$

is supposed to be twice differentiable and satisfy

(7) $$\overset{ˇ}{G}\left(t,\cdot \right)\in L^{1}\left({I\;R}^{+}\right)\cap C^{2}\left({I\;R}^{+}\right)$$

for all t∈IR, together with the further prescriptions on the signature of its derivatives that derive from the physics of the model (see [19], assumptions M1–M4). In unidimensional problems, the relaxation kernel reduces to a scalar function G, and its derivatives must satisfy

(8) $$G\left(t,s\right)>0,\;G_{s}\left(t,s\right)\leq 0,\;G_{ss}\left(t,s\right)\geq 0,\;\left(t,s\right)\in I\;R\times {I\;R}^{+},$$

(9) $$G_{t}\left(t,s\right)+G_{s}\left(t,s\right)\leq 0,\;G_{ts}\left(t,s\right)+G_{ss}\left(t,s\right)\geq 0\;\left(t,s\right)\in I\;R\times {I\;R}^{+}.$$

Since G(t,s) reduces to G(s) when aging is neglected, assumptions (8) correspond to classical Graffi’s conditions, whereas (9) boils down to (8).

A typical example of an aging memory kernel is given by

$$G\left(t,s\right)=G_{\infty }\left(t\right)+\exp\left(-\frac{s}{\varepsilon (t)}\right),$$

where $G_{\infty },\varepsilon \in C^{1}\left(I\;R,{I\;R}^{+}\right)$ satisfy

$$G_{\infty }^{\prime }\left(t\right)\leq 0,\;\varepsilon^{\prime }\left(t\right)\leq 0,\;\forall t\in I\;R.$$

It is easy to verify that assumptions (8), (9) are complied. The corresponding stress–strain relation describes a standard linear solid with a damping component that ages, losing effectiveness.

Another example is obtained by a suitable rescaling of a (non-negative) non-increasing function $\overset{ˇ}{G}\in L^{1}\left({I\;R}^{+}\right)\cap C^{1}\left({I\;R}^{+}\right)$. Given $t_{0}>0$ and $\varepsilon \in C^{1}\left(I\;R,{I\;R}^{+}\right)$ satisfying $\varepsilon^{\prime }\left(t\right)\leq 0$, ∀t∈IR, we define

(10) $$\overset{ˇ}{G}\left(t,s\right)=\frac{1}{\varepsilon (t)}\;\overset{ˇ}{G}\left(\frac{s}{\varepsilon (t)}\right)$$

In particular, for all t∈IR, we obtain

$$\int_{0}^{\infty }\overset{ˇ}{G}\left(t,s\right)\;d\;s=\int_{0}^{\infty }\overset{ˇ}{G}\left(s\right)\;d\;s=\mu <\infty .$$

**Figure 1:**
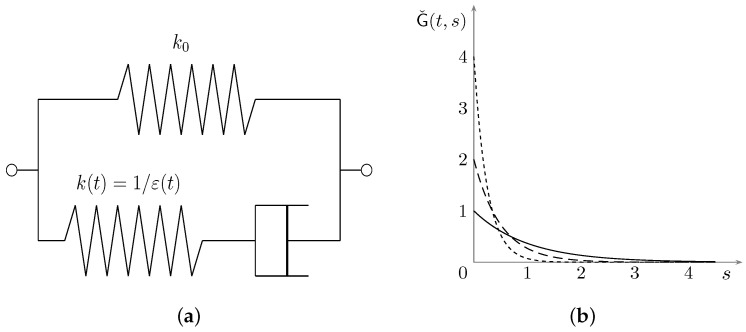
(**a**) The standard solid model with an aging spring component. (**b**) Plot of the memory kernel $\overset{ˇ}{G}$ at t=100 s (solid), t=200 s (dashed), t=400 s (short dashed).

Accordingly, if ε(t)→0 as t→∞, we obtain the distributional convergence

$$\lim_{t\to \infty }\overset{ˇ}{G}\left(t,\cdot \right)=\mu \delta_{0},$$

where $\delta_{0}$ denotes the Dirac mass at $0^{+}$. As proved in [22] and depicted in Figure 1a, (10) describes aging as a transition from viscoelasticity with long memory (standard linear solid) to viscoelasticity with short memory (Kelvin–Voigt model). For definiteness, we take $\overset{ˇ}{G}\left(s\right)=e^{-s}$ and ε(t)=α/t, α>0, in which case (see Figure 1b where α=100)

(11) $$\overset{ˇ}{G}\left(t,s\right)=\frac{t}{\alpha }\exp\left(-\frac{st}{\alpha }\right)$$

The corresponding relaxation kernel $G=G_{\infty }+\overset{ˇ}{G}$ complies with assumptions (8), (9) provided that $t\geq \sqrt{2\alpha }$ and $G_{\infty }^{\prime }\leq 0$.

## 3. The Problem

The problem under investigation models materials which couple a time-dependent viscoelastic behavior with a magnetic one. For the sake of simplicity, the body here considered is one-dimensional.

The magneto-elastic interaction is modeled according to [14,15,16] while the magneto-viscoelastic regular behavior is the one given in [17,18].

**Figure 2:**
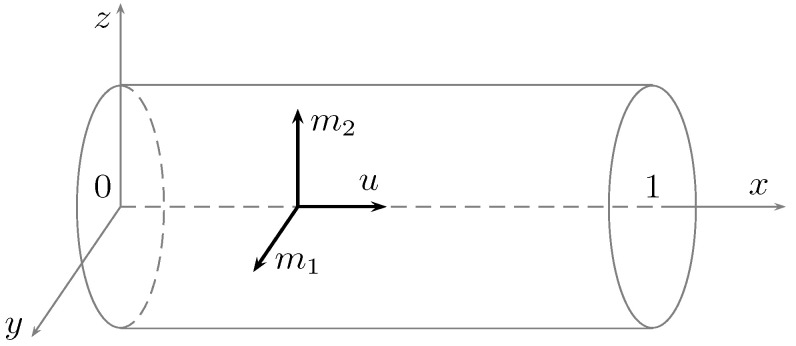
A picture of the problem under consideration: the cylindrical sample is assumed to have a negligible radius compared to its length.

Let Ω=(0,1) and Q:=Ω×(0,T), T>0. The system to study is given by

(12) $$\left\{\begin{array}{c} u_{tt}-G\left(t,0\right)u_{xx}-\int_{0}^{t}G_{s}\left(t,s\right)u_{xx}\left(t-s\right)ds-\frac{\lambda }{2}{\left(L\left(m\right)\cdot m\right)}_{x}=f\\ \;\;\mathrm{in}\;Q\\ m_{t}+\delta^{-1}\left[|m{|}^{2}-1\right]m+\lambda L\left(m\right)u_{x}-m_{xx}=0, \end{array}\right.$$

together with the initial and boundary conditions

(13) $$u\left(\cdot ,0\right)=u_{0},\;\;\;u_{t}\left(\cdot ,0\right)=u_{1},\;m\left(\cdot ,0\right)=m_{0},\;\;\;\left|m_{0}\right|=1,\;\mathrm{in}\;\;\Omega \;,$$

(14) $$u\left(0,\cdot \right)=u\left(1,\cdot \right)=0,\;m_{x}\left(0,\cdot \right)=m_{x}\left(1,\cdot \right)=0\;in\;\left(0,T\right)\;,$$

where the displacement is u≡(u,0,0), since the direction of the conductor is here identified with the *x*-axis, and the magnetization vector, M≡(0,m), $m=(m_{1},m_{2})$, is orthogonal to the conductor itself (see Figure 2). All fields $u,m_{1},m_{2}$ are functions of (x,t)∈Ω×(0,T). In addition, the linear operator L is defined by $L\left(m\right)=\left(m_{2},m_{1}\right)$ and λ,δ are positive parameters. Finally, the term *f* is given by the sum of an external (longitudinal) force and the contribution of the deformation history up to the initial time,

$$\int_{t}^{\infty }G_{s}\left(t,s\right)u_{xx}\left(t-s\right)ds=-\int_{-\infty }^{0}G_{\tau }\left(t,\tau \right)u_{xx}\left(\tau \right)d\tau .$$

Moreover, we assume

(15) $$u_{0}\in H_{0}^{1}\left(\Omega \right),\;u_{1}\in L^{2}\left(\Omega \right),\;m_{0}\in H^{1}\left(\Omega \right),\;f\in L^{2}\left(Q\right).$$

In addition, the kernel, G:T→IR, $T={\left[0,T\right]}^{2}$, is supposed to satisfy (7) together with (8) and (9).

Taking into account only the mechanical aspects of the problem, the following linear integro-differential equation in Q is considered

(16) $${\tilde{u}}_{tt}\left(t\right)-G\left(t,0\right){\tilde{u}}_{xx}\left(t\right)-\int_{0}^{t}G_{s}\left(t,s\right){\tilde{u}}_{xx}\left(t-s\right)ds=F\left(t\right).$$

The initial and boundary conditions, in turn, are

(17) $$\begin{array}{cc} \; & \tilde{u}\left(\cdot ,0\right)=u_{0},\;\;\;{\tilde{u}}_{t}\left(\cdot ,0\right)=u_{1},\;\mathrm{in}\;\Omega \\ \; & \tilde{u}\left(0,\cdot \right)=\bar{u}\left(1,\cdot \right)=0,\;\;\;\mathrm{in}\;\left(0,T\right)\;. \end{array}$$

Note that, as proved in [22], problem (16)–(17) admits a unique strong solution. In particular, the following result holds.

**Lemma** **1.** *Denote by $\tilde{u}$ the unique solution admitted to the problem* (16)–(17)*with $F\in L^{2}\left(Q\right)$. Then, for all t∈[0,T], the following estimate is obtained*

(18) $$\begin{array}{cc} \; & \frac{1}{2}\int_{\Omega }G\left(t,t\right)\;|{\tilde{u}}_{x}{\left(t\right)|}^{2}\;dx+\frac{1}{2}\int_{\Omega }{\left|{\tilde{u}}_{t}\left(t\right)\right|}^{2}\;dx\\ \; & \leq \frac{1}{2}\int_{\Omega }G\left(0,0\right)\;|{\tilde{u}}_{0x}{|}^{2}\;dx+\frac{1}{2}\int_{\Omega }{\left|u_{1}\right|}^{2}\;dx+\int_{\Omega }\int_{0}^{t}F\left(\tau \right)\;{\tilde{u}}_{\tau }\left(\tau \right)\;dx\;d\tau . \end{array}$$

**Proof.**  First of all, add and subtract to Equation (16) the term

$$\int_{0}^{t}G_{s}\left(t,s\right){\tilde{u}}_{xx}\left(t\right)ds= [G(t,t)-G(t,0)]{\tilde{u}}_{xx}\left(t\right)\;.$$

The result can be written in the equivalent form

(19) $${\tilde{u}}_{tt}-G\left(t,t\right){\tilde{u}}_{xx}+\int_{0}^{t}G_{s}\left(t,s\right) [{\tilde{u}}_{xx}\left(t\right)-{\tilde{u}}_{xx}\left(t-s\right)]ds=F.$$

when Equation (19) is multiplied by ${\tilde{u}}_{t}$, after integration over Ω, it follows

(20) $$\begin{array}{c} \frac{1}{2}\frac{d}{dt}\int_{\Omega }{\left|{\tilde{u}}_{t}\left(t\right)\right|}^{2}dx\;+\;\int_{\Omega }G\left(t,t\right){\tilde{u}}_{x}\left(t\right)\;{\tilde{u}}_{xt}\left(t\right)\;dx\;+\\ -\int_{\Omega }{\tilde{u}}_{xt}\left(t\right)\;\int_{0}^{t}G_{s}\left(t,s\right)\; [{\tilde{u}}_{x}\left(t\right)-{\tilde{u}}_{x}\left(t-s\right)]ds\;dx=\int_{\Omega }F{\tilde{u}}_{t}\left(t\right)\;dx. \end{array}$$

Since $\frac{d}{dt}G\left(t,t\right)=\left[G_{t}+G_{s}\right]\left(t,s\right){|}_{s=t}$, it follows

(21) $$\begin{array}{c} \frac{1}{2}\frac{d}{dt}\int_{\Omega }|{\tilde{u}}_{t}{|}^{2}dx\;+\;\frac{1}{2}\frac{d}{dt}\int_{\Omega }G\left(t,t\right)|{\tilde{u}}_{x}{|}^{2}\;dx=\frac{1}{2}\int_{\Omega }\left[G_{t}+G_{s}\right]\left(t,t\right){\left|{\tilde{u}}_{x}\right|}^{2}\;dx\\ +\int_{\Omega }\;\int_{0}^{t}G_{s}\left(t,s\right){\tilde{u}}_{xt}\left(t\right) [{\tilde{u}}_{x}\left(t\right)-{\tilde{u}}_{x}\left(t-s\right)]ds\;dx+\int_{\Omega }F{\tilde{u}}_{t}\;dx. \end{array}$$

Now, we observe that

$$\frac{\partial }{\partial t}\left[{\tilde{u}}_{x}\left(t\right)-{\tilde{u}}_{x}\left(t-s\right)\right]={\tilde{u}}_{xt}\left(t\right)-\frac{\partial }{\partial s} [{\tilde{u}}_{x}\left(t\right)-{\tilde{u}}_{x}\left(t-s\right)],\;0\leq s\leq t\leq T,$$

and then

$${\tilde{u}}_{xt}\left(t\right)\left[{\tilde{u}}_{x}\left(t\right)-{\tilde{u}}_{x}\left(t-s\right)\right]=\frac{1}{2} [\frac{\partial }{\partial t}|{\tilde{u}}_{x}\left(t\right)-{\tilde{u}}_{x}{\left(t-s\right)|}^{2}+\frac{\partial }{\partial s}{\left|{\tilde{u}}_{x}\left(t\right)-{\tilde{u}}_{x}\left(t-s\right)\right|}^{2}]\;\;.$$

Substitution within the double integral in (21) gives

$$\begin{array}{cc} & \int_{\Omega }\int_{0}^{t}G_{s}\left(t,s\right)\;{\tilde{u}}_{xt}\left[{\tilde{u}}_{x}\left(t\right)-{\tilde{u}}_{x}\left(t-s\right)\right]\;dxds\\ & =\frac{1}{2}\int_{0}^{t}\int_{\Omega }G_{s}\left(t,s\right)\;\frac{\partial }{\partial t}{\left|{\tilde{u}}_{x}\left(t\right)-{\tilde{u}}_{x}\left(t-s\right)\right|}^{2}dxds\\ & \frac{1}{2}\int_{0}^{t}\int_{\Omega }G_{s}\left(t,s\right)\;\frac{\partial }{\partial s}{\left|{\tilde{u}}_{x}\left(t\right)-{\tilde{u}}_{x}\left(t-s\right)\right|}^{2}dxds\\ & =\frac{1}{2}\frac{d}{dt}\int_{0}^{t}\int_{\Omega }G_{s}\left(t,s\right){\left|{\tilde{u}}_{x}\left(t\right)-{\tilde{u}}_{x}\left(t-s\right)\right|}^{2}dxds\\ & -\frac{1}{2}\int_{\Omega }\int_{0}^{t}\left[G_{st}+G_{ss}\right]\left(t,s\right){\left|{\tilde{u}}_{x}\left(t\right)-{\tilde{u}}_{x}\left(t-s\right)\right|}^{2}dxds. \end{array}$$

Taking into account the sign conditions (8)–(9), from (21), we obtain

(22) $$\begin{array}{c} \frac{1}{2}\frac{d}{dt}\int_{\Omega }|{\tilde{u}}_{t}{|}^{2}dx\;+\;\frac{1}{2}\frac{d}{dt}\int_{\Omega }G\left(t,t\right){\left|{\tilde{u}}_{x}\right|}^{2}\;dx\leq \\ \frac{1}{2}\frac{d}{dt}\int_{0}^{t}\int_{\Omega }G_{s}\left(t,s\right){\left|{\tilde{u}}_{x}\left(t\right)-{\tilde{u}}_{x}\left(t-s\right)\right|}^{2}dxds+\int_{\Omega }F{\tilde{u}}_{t}\;dx. \end{array}$$

Integration over time, in the range (0,t), t∈(0,T), taking into account the sign conditions (8) implies (18) and, hence, completes the proof. ☐

According to (8)${}_{1}$, let

$$g_{0}=\min_{t\in [0,T]}G\left(t.t\right)>0,\;g_{1}=\max_{t\in [0,T]}G\left(t.t\right)>0.$$

As a consequence of (18)

$$\begin{array}{cc} \; & g_{0}\int_{\Omega }|{\tilde{u}}_{x}{\left(t\right)|}^{2}\;dx+\int_{\Omega }|{\tilde{u}}_{t}{\left(t\right)|}^{2}\;dx\leq g_{1}\int_{\Omega }|{\tilde{u}}_{0x}{|}^{2}\;dx+\int_{\Omega }{\left|u_{1}\right|}^{2}\;dx\\ \; & +\int_{\Omega }\int_{0}^{t}{\left|F\left(\tau \right)\right|}^{2}dx\;d\tau +\int_{\Omega }\int_{0}^{t}{\left|{\tilde{u}}_{\tau }\left(\tau \right)\right|}^{2}dx\;d\tau , \end{array}$$

which, on application of Gronwall’s Lemma, implies

(23) $$∥\tilde{u}{\left(t\right)∥}_{H_{0}^{1}\left(\Omega \right)}^{2}+{∥{\tilde{u}}_{t}\left(t\right)∥}_{L^{2}\left(\Omega \right)}^{2}\leq Ce^{T}\;,$$

where $C=\hat{C}(∥u_{0}{∥}_{H_{0}^{1}\left(\Omega \right)},∥u_{1}{∥}_{L^{2}\left(\Omega \right)}{,∥F∥}_{L^{2}\left(Q\right)})>0$. The estimate thus obtained is needed subsequently, together with the following one.

**Lemma** **2.** *Let (u,m) be a solution admitted to the problem* (12)–(15)*. Then, the following estimate holds*

(24) $$\begin{array}{c} \;\int_{\Omega }G\left(t,t\right)|u_{x}{|}^{2}\;dx+\int_{\Omega }|u_{t}{|}^{2}\;dx+2\int_{0}^{t}\int_{\Omega }|m_{t}{|}^{2}\;dx\;ds+\int_{\Omega }{\left|m_{x}\right|}^{2}\;dx\\ +\lambda \int_{\Omega }\left[L\left(m\right)\cdot m\right]u_{x}\;dx+\frac{1}{2\delta }\int_{\Omega }{\left(|m\right|}^{2}{-1)}^{2}\;dx\leq 2\int_{0}^{t}\int_{\Omega }fu_{t}dx\\ +\int_{\Omega }G\left(0,0\right)\;|u_{0x}{|}^{2}\;dx+\lambda \int_{\Omega }\left[L\left(m_{0}\right)\cdot m_{0}\right]u_{0x}dx+\int_{\Omega }|m_{0x}{|}^{2}\;dx+\int_{\Omega }{\left|u_{1}\right|}^{2}\;dx\;\;. \end{array}$$

**Proof.**  Taking the scalar product of (12)${}_{2}$ with $m_{t}$ and then integrating over Ω, it follows

$$\;\;\int_{\Omega }{\left|m_{t}\right|}^{2}\;dx+\frac{1}{4\delta }\frac{d}{dt}\int_{\Omega } [{\left({\left|m\right|}^{2}-1\right)}^{2}+2\delta |m_{x}{|}^{2}]dx+\lambda \int_{\Omega }\left(L\left(m\right)\cdot m_{t}\right)u_{x}=0$$

and hence, after integration over (0,t), since $|m_{0}|=1$. Then, it allows writing (13),

(25) $$\begin{array}{c} \int_{0}^{t}\int_{\Omega }{\left|m_{t}\right|}^{2}\;dx+\frac{1}{4}\int_{\Omega }\frac{{\left({\left|m\right|}^{2}-1\right)}^{2}}{\delta }\;dx+\frac{1}{2}\int_{\Omega }|m_{x}{|}^{2}\;dx\\ +\lambda \int_{0}^{t}\int_{\Omega }\left[L\left(m\right)\cdot m_{t}\right]u_{x}dx=\frac{1}{2}\int_{\Omega }|{m_{0}}_{x}{|}^{2}\;dx \end{array}$$

Now, multiplying (12)${}_{1}$ by $u_{t}$, integrating over Ω and following the lines of the proof of Lemma 1, is obtained

(26) $$\begin{array}{c} \frac{1}{2}\int_{\Omega }G\left(t,t\right)\;|u_{x}{\left(t\right)|}^{2}\;dx+\frac{1}{2}\int_{\Omega }{\left|u_{t}\left(t\right)\right|}^{2}\;dx\leq \frac{1}{2}\int_{\Omega }G\left(0,0\right)\;{\left|u_{0x}\right|}^{2}\;dx\\ +\frac{1}{2}\int_{\Omega }{\left|u_{1}\right|}^{2}\;dx+\int_{\Omega }\int_{0}^{t}fu_{t}\;dx\;ds-\frac{\lambda }{2}\int_{\Omega }\int_{0}^{t} [L(m)\cdot m]\;u_{xt}\;dx\;ds. \end{array}$$

Since

$$L\left(m\right)\cdot m_{t}=\frac{1}{2}\frac{d}{dt}\left[L\left(m\right)\cdot m\right],$$

doubling the sum of (25) and (26), the inequality (24) is obtained, and the proof is completed. ☐

As a consequence of Lemma 2, the following estimates of displacement and magnetic field are proved.

**Lemma** **3.** *Let (u,m) be a solution admitted to problem* (12)–(15) *with conditions* (7)–(9)*. If δ is small enough, then*

(27) $$\begin{array}{c} \int_{\Omega }|u_{x}{|}^{2}\;dx\leq C_{1},\;\int_{\Omega }{\left|u_{t}\right|}^{2}\;dx\leq C_{2},\;\int_{\Omega }|m_{x}{|}^{2}\;dx\leq C_{3}\\ \int_{Q}{\left|m_{t}\right|}^{2}\;dxdt\leq C_{4},\;\int_{\Omega }\left|{\left|m\right|}^{2}-1\right|\;dx\leq \sqrt{\delta }C_{5} \end{array}$$

*where $C_{k},k=1,...,5$, depend only on $T,u_{0},u_{1},m_{0},f$ and |Ω|.*

**Proof.**  In considering inequality (24) proved in Lemma 2, observe that

$$\left|\int_{\Omega }\left(L\left(m\right)\cdot m\right)u_{x}dx\right|\leq \int_{\Omega }{\left|m\right|}^{2}\left|u_{x}\right|dx.$$

Furthermore,

$$\begin{array}{c} \int_{\Omega }{\left|m\right|}^{2}|u_{x}|dx=\sqrt{\delta }\int_{\Omega }\frac{\left({\left|m\right|}^{2}-1\right)}{\sqrt{\delta }}|u_{x}|\;dx+\int_{\Omega }\left|u_{x}\right|dx\leq \\ \frac{\sqrt{\delta }}{2}\int_{\Omega }\frac{{\left({\left|m\right|}^{2}-1\right)}^{2}}{\delta }dx+\frac{1}{2}\left(\sqrt{\delta }+\sigma \right)\int_{\Omega }|u_{x}{|}^{2}dx+\frac{1}{2\sigma }\left|\Omega \right|\;, \end{array}$$

for any σ>0. Note that 1/δ plays the role of a penalty coefficient (see [16]); in addition, σ and δ can be chosen small enough in such a way that

(28) $$2\lambda \sqrt{\delta }<1\;,\;\;\;\lambda \left(\sqrt{\delta }+\sigma \right)<g_{0}:=\min_{t\in [0,T]}G\left(t.t\right)$$

hence, (24) leads to

(29) $$\begin{array}{c} \frac{1}{2}\;g_{0}\int_{\Omega }|u_{x}{|}^{2}\;dx+\int_{\Omega }|u_{t}{|}^{2}\;dx+\int_{\Omega }|m_{x}{|}^{2}\;dx+2\int_{0}^{t}\int_{\Omega }{\left|m_{t}\right|}^{2}\;dx\;ds\\ +\frac{1}{4}\int_{\Omega }\frac{{\left(|m\right|}^{2}{-1)}^{2}}{\delta }\;dx\leq 2\int_{0}^{t}\int_{\Omega }fu_{t}dxds+C_{0}\;. \end{array}$$

where the dependence on |Ω| and on initial data $u_{0},u_{1},m_{0}$ is included within the constant $C_{0}$. Noticing that

$$\begin{array}{c} 2\left|\int_{0}^{t}\int_{\Omega }fu_{t}\;dx\;ds\right|\leq \int_{0}^{t}\int_{\Omega }|u_{t}{|}^{2}\;dx\;ds+\int_{0}^{t}\int_{\Omega }{\left|f\right|}^{2}\;dx\;ds,\\ \int_{\Omega }{\left(|m\right|}^{2}{-1)}^{2}\;dx\geq {\left(\int_{\Omega }\frac{{\left||m\right|}^{2}-1|}{\sqrt{\left|\Omega \right|}}\;dx\right)}^{2}, \end{array}$$

by letting

$$E\left(t\right)=\frac{1}{2}\;g_{0}\int_{\Omega }|u_{x}{|}^{2}\;dx+\int_{\Omega }{\left|u_{t}\right|}^{2}\;dx+\int_{\Omega }|m_{x}{|}^{2}\;dx+{\left(\int_{\Omega }\frac{{\left||m\right|}^{2}-1|}{2\sqrt{\delta |\Omega |}}\;dx\right)}^{2},$$

via (29)

(30) $$E\left(t\right)-\int_{0}^{t}E\left(\tau \right)d\tau +2\int_{0}^{t}\int_{\Omega }|m_{t}{|}^{2}\;dx\;ds\leq C(T,u_{0},u_{1},m_{0},f,|\Omega |),isobtained.$$

Hence, recalling Gronwall Lemma, for any t∈(0,T), it follows that

$$E\left(t\right),\int_{0}^{t}E\left(\tau \right)d\tau ,\int_{0}^{t}\int_{\Omega }|m_{t}{|}^{2}\;dx\;ds\leq \tilde{C}(T,u_{0},u_{1},m_{0},f,|\Omega |)\;,$$

which completes the proof. Indeed, due to the expression of E, all the inequalities (27) are proved. ☐

## 4. An Existence and Uniqueness Result

This section is concerned about weak solutions to the non-linear integro-differential problem (12)–(15). Specifically, under conditions (7)–(9) the problem is proved to admit a unique weak solution. The result is first established in a small time interval using a fixed-point theorem and then extended to any interval [0,T] by exploiting the previous a priori estimates. Note that the result, following the procedure devised in [18], can be generalized in the case of a three-dimensional magneto-viscoelastic material. Indeed, the a priori estimates of Lemmas 2.1 and 2.2 can be easily extended.

First of all, a local result is established.

**Lemma** **4.** Let us take the same assumptions as in Lemma 3. Then, depending on the data of the problem, $u_{0}$, $u_{1}$, $m_{0}$ and f, there exists $t^{*}\in \left(0,T\right]$ such that the initial-boundary value problem (12)–(15)*subject to conditions* (7)–(9), has one and only one solution (u,m) in $\bar{\Omega }\times \left[0,t^{*}\right]$ that satisfies:

- $u\in C^{0}\left(\left[0,t^{*}\right];H_{0}^{1}\left(\Omega \right)\right)\cap C^{1}\left(\left[0,t^{*}\right];L^{2}\left(\Omega \right)\right)$;
- $m\in C^{0}\left(\left[0,t^{*}\right];H^{1}\left(\Omega \right)\right)$;
- $m_{t}\in L^{2}\left(0,t^{*};L^{2}\left(\Omega \right)\right)$.

**Proof.**  For simplicity, we introduce the following notations. The $L^{2}\left(\Omega \right)$-norm is denoted by ∥·∥, the $L^{\infty }\left(\Omega \right)$-norm by ${∥\cdot ∥}_{\infty }$ and the $H^{1}\left(\Omega \right)$-norm by ${∥\cdot ∥}_{1}$. Let $M_{1},M_{2}$ be any pair of positive constants and let h∈(0,T]. Moreover, define

$$C=C^{0}\left(\left[0,h\right];H_{0}^{1}\left(\Omega \right)\right)\times C^{0}\left(\left[0,h\right];L^{2}\left(\Omega \right)\right).$$

Let $B^{h}=B_{1}^{h}\times B_{2}^{h}$ be the convex set such that

$$B_{1}^{h}\equiv \left\{\left(u,u_{t}\right)\in C:\;u\left(0\right)=u_{0},\;u_{t}\left(0\right)=u_{1},\;\underset{\left[0,h\right]}{\sup} [{∥u∥}_{1}^{2}+{∥u_{t}∥}^{2}]\leq M_{1}\right\}$$

$$B_{2}^{h}\equiv \left\{\left(m,m_{t}\right)\in C:\;|m\left(0\right)\left|=\right|m_{0}|=1,m_{x}{|}_{x=0,1}=0,\;\underset{\left[0,h\right]}{\sup}{∥m∥}_{1}^{2}+\int_{0}^{h}{∥m_{t}∥}^{2}dt\leq M_{2}\right\}.$$

Let $\left(\bar{u},\bar{m}\right)\in B^{h}$ and consider the following linear problem

(31) $$\left\{\begin{array}{c} u_{tt}=G\left(t,0\right)u_{xx}+\int_{0}^{t}G_{s}\left(t,s\right)u_{xx}\left(t-s\right)ds+\frac{\lambda }{2}\;{\left(L\left(\bar{m}\right)\cdot \bar{m}\right)}_{x}+f,\\ m_{t}=m_{xx}-\lambda L\left(\bar{m}\right){\bar{u}}_{x}-\delta^{-1}\left(\right|\bar{m}{|}^{2}-1)\bar{m}, \end{array}\right.$$

To start with, observe that system (31) is uncoupled and, for any arbitrarily fixed $\left(\bar{u},\bar{m}\right)\in B^{h}$, each equation admits a classical results of existence and uniqueness. Hence, the linear problem (31), subject to conditions (13)–(15) and (7)–(9), has a unique solution (u,m) such that $u\in B_{1}^{h}$ and $m\in B_{2}^{h}$. Moreover, as a consequence of Lemmas 2 and 3, such a solution satisfies the following estimates

(*i*) $∥u_{t}{\left(t\right)∥}^{2}+{∥u_{x}\left(t\right)∥}^{2}\leq \left(C_{1}\;+\;t\;K_{1}\;\right)e^{2t},\;t\in \left[0,h\right]$,
(*ii*) $\int_{0}^{t}∥m_{\tau }{\left(\tau \right)∥}^{2}d\tau +{∥m\left(t\right)∥}_{H^{1}\left(\Omega \right)}^{2}\leq \left(C_{2}\;+\;t\;K_{2}\;\right)e^{t},\;t\in \left[0,h\right]$,

where $K_{1},K_{2}$ are positive constants which depend on both $M_{1}$ and $M_{2}$ and

$$C_{1}={\tilde{C}}_{1}(∥u_{0}{∥}_{1},∥u_{1}{∥,∥f∥}_{L^{2}\left(Q\right)})>0,\;C_{2}={\tilde{C}}_{2}(∥m_{0}{∥}_{1})>0.$$

The estimates (i) and (ii) imply that if we set $M_{1}=2C_{1}$ and, $M_{2}=2C_{2}$, then, taking t small enough, the solution $\left(u,m\right)\in B^{h}$ and hence $\left(\bar{u},\bar{m}\right)\mapsto \left(u,m\right)$ maps $B^{h}$ into itself. Banach fixed point theorem, once the mapping $\left(\bar{u},\bar{m}\right)\mapsto \left(u,m\right)$ is proved to be a contraction, allows proving the existence of the solution. Consider $\left(\bar{u},\bar{m}\right)$ and $\left(\bar{\bar{u}},\bar{\bar{m}}\right)$ two fixed pairs in $B^{h}$ and denote by (u,m) and $\left(\tilde{u},\tilde{m}\right)$ the corresponding solutions of the linearized problem (31). To obtain the contraction property for *t* small enough, we need to prove that the differences

$$\left(z,q\right)=\left(u-\tilde{u},m-\tilde{m}\right),\;\left(\bar{z},\bar{q}\right)=\left(\bar{u}-\bar{\bar{u}},\bar{m}-\bar{\bar{m}}\right)$$

satisfy the following inequality

(*iii*) $∥z_{t}{\left(t\right)∥}^{2}+{∥z\left(t\right)∥}_{1}^{2}+\int_{0}^{t}∥q_{\tau }{\left(\tau \right)∥}^{2}d\tau +{∥q\left(t\right)∥}_{1}^{2}$
  $\leq t\;K_{3}\;e^{t}\;\underset{\left[0,h\right]}{\sup}\left(∥{\bar{z}}_{t}{\left(t\right)∥}^{2}+∥\bar{z}{\left(t\right)∥}_{1}^{2}+\int_{0}^{t}∥{\bar{q}}_{t}{∥}^{2}d\tau +{∥\bar{q}\left(t\right)∥}_{1}^{2}\right),$

where $K_{3}>0$ depends on $M_{1}$, $M_{2}$.

Inequality (iii) can be proved analogously to estimates established above. First, we consider Equation (31)${}_{1}$ for both *u* and $\tilde{u}$. If we then subtract them from each other, we obtain

$$z_{tt}=G\left(t,0\right)z_{xx}+\int_{0}^{t}G_{s}\left(t,s\right)z_{xx}\left(t-s\right)ds+\lambda \;L\left(\bar{m}\right)\cdot {\bar{m}}_{x}-\lambda \;L\left(\bar{\bar{m}}\right)\cdot {\bar{\bar{m}}}_{x}.$$

Operating on the first terms of this equation as in Lemma 1, multiplication by $z_{t}$ and integration over Ω gives

$$\begin{array}{cc} & \frac{1}{2}\frac{d}{dt}\{∥z_{t}{\left(t\right)∥}^{2}+G\left(t,t\right)∥z_{x}{\left(t\right)∥}^{2}-\int_{0}^{t}G_{s}\left(t,s\right)∥z_{x}\left(t\right)-z_{x}\left(t-s\right){∥}^{2}ds\}\\ & \leq \lambda \int_{\Omega }\left\{L\left(\bar{m}\left(t\right)\right)\cdot {\bar{m}}_{x}\left(t\right)-L\left(\bar{\bar{m}}\left(t\right)\right)\cdot {\bar{\bar{m}}}_{x}\left(t\right)\right\}z_{t}\left(t\right)dx\\ & =\lambda \int_{\Omega }L\left(\bar{m}\left(t\right)\right)\cdot {\bar{q}}_{x}\left(t\right)z_{t}\left(t\right)dx+\lambda \int_{\Omega }L\left(\bar{q}\left(t\right)\right)\cdot {\bar{\bar{m}}}_{x}\left(t\right)z_{t}\left(t\right)dx\\ & \leq \lambda \underset{\left[0,h\right]}{\sup}∥L\left(\bar{m}\left(t\right)\right){∥}_{\infty }∥{\bar{q}}_{x}\left(t\right)∥\;∥z_{t}\left(t\right)∥+\lambda \underset{\left[0,h\right]}{\sup}∥L\left(\bar{q}\left(t\right)\right){∥}_{\infty }∥{\bar{\bar{m}}}_{x}\left(t\right)∥\;∥z_{t}\left(t\right)∥\\ & \leq 2\lambda \sqrt{M_{2}}∥{\bar{q}}_{x}\left(t\right)∥\;∥z_{t}\left(t\right)∥\leq \frac{1}{2}∥z_{t}{\left(t\right)∥}^{2}+2\lambda^{2}M_{2}{∥\bar{q}\left(t\right)∥}_{H^{1}\left(\Omega \right)}^{2}. \end{array}$$

Let

$$E\left(t\right)=∥z_{t}{\left(t\right)∥}^{2}+G\left(t,t\right)∥z_{x}{\left(t\right)∥}^{2}-\int_{0}^{t}G_{s}\left(t,s\right){∥z_{x}\left(t\right)-z_{x}\left(t-s\right)∥}^{2}ds$$

and note that E(0)=0. Moreover, since $G_{s}\left(t,s\right)\leq 0$ and $G\left(t,t\right)\geq g_{0}>0$, it follows E(t)≥0 for all t∈(0,T). We can then apply the previous inequality so obtaining

$$\frac{\;d}{dt}\left[e^{-t}E\left(t\right)\right]\leq 4\lambda^{2}M_{2}∥\bar{q}{\left(t\right)∥}_{H^{1}\left(\Omega \right)}^{2}e^{-t}\leq 4\lambda^{2}M_{2}\underset{\left[0,h\right]}{\sup}{∥\bar{q}\left(t\right)∥}_{H^{1}\left(\Omega \right)}^{2}\;,$$

and therefore, it follows

$$∥z_{t}{\left(t\right)∥}^{2}+g_{0}∥z_{x}{\left(t\right)∥}^{2}\leq 4\lambda^{2}M_{2}\;te^{t}\underset{\left[0,h\right]}{\sup}{∥\bar{q}\left(t\right)∥}_{H^{1}\left(\Omega \right)}^{2}.$$

Letting $\alpha =\max\{g_{0}^{-1},1\}$, we finally have

(32) $$∥z_{t}{\left(t\right)∥}^{2}+∥z_{x}{\left(t\right)∥}^{2}\leq 4\alpha \lambda^{2}M_{2}\;te^{t}\underset{\left[0,h\right]}{\sup}{∥\bar{q}\left(t\right)∥}_{H^{1}\left(\Omega \right)}^{2}.$$

Taking into account (31)${}_{2}$ for both m and $\tilde{m}$ allows writing

(33) $$q_{t}=q_{xx}-\lambda L\left(\bar{m}\right){\bar{z}}_{x}+\lambda L\left(-\bar{q}\right){\bar{\bar{u}}}_{x}-\delta^{-1}\left(\right|\bar{m}{|}^{2}-1)\bar{q}+\delta^{-1}\left(\right|\bar{\bar{m}}{|}^{2}-|\bar{m}{|}^{2})\bar{\bar{m}}.$$

Multiplication of this equation by $q_{t}$ and the subsequent integration over Ω implies

$$\begin{array}{cc} ∥q_{t}{∥}^{2} & +\frac{1}{2}\frac{d}{dt}{∥q_{x}∥}^{2}=-\lambda \int_{\Omega }L\left(\bar{m}\right)\cdot q_{t}\;{\bar{z}}_{x}dx+\lambda \int_{\Omega }L\left(-\bar{q}\right)\cdot q_{t}\;{\bar{\bar{u}}}_{x}dx\\ & -\delta^{-1}\int_{\Omega }\left(\right|\bar{m}{|}^{2}-1)\bar{q}\;\cdot q_{t}\;dx-\delta^{-1}\int_{\Omega }\bar{q}\cdot \left(\bar{\bar{m}}+\bar{m}\right)\left[\;\bar{\bar{m}}\cdot q_{t}\right]dx\\ & \leq \lambda \sqrt{2M_{2}}∥q_{t}∥∥{\bar{z}}_{x}∥+\lambda \underset{0\leq t\leq h}{\sup}∥\bar{q}{∥}_{\infty }∥q_{t}∥∥{\bar{\bar{u}}}_{x}∥\\ & +\delta^{-1}\left(2M_{2}+1\right)∥\bar{q}∥∥q_{t}∥+\delta^{-1}4M_{2}∥\bar{q}∥∥q_{t}∥\\ & \leq \{\lambda \sqrt{2M_{2}}∥{\bar{z}}_{x}∥+\left[\lambda \sqrt{2M_{1}}+\delta^{-1}\left(2M_{2}+1\right)+\delta^{-1}4M_{2}\right]\;∥\bar{q}{∥}_{H^{1}\left(\Omega \right)}\}∥q_{t}∥\\ & \leq 2\lambda^{2}M_{2}∥{\bar{z}}_{x}{∥}^{2}+{\left[\lambda \sqrt{2M_{1}}+\delta^{-1}\left(2M_{2}+1\right)+\delta^{-1}4M_{2}\right]}^{2}\;∥\bar{q}{∥}_{H^{1}\left(\Omega \right)}^{2}+\frac{1}{2}{∥q_{t}∥}^{2}. \end{array}$$

Accordingly, we obtain

$$∥q_{t}{\left(t\right)∥}^{2}+\frac{d}{dt}∥q_{x}{\left(t\right)∥}^{2}\leq K_{1}\underset{\left[0,h\right]}{\sup}(∥{\bar{z}}_{x}{\left(t\right)∥}^{2}+∥\bar{q}\left(t\right){∥}_{H^{1}\left(\Omega \right)}^{2})\;,$$

where

$$K_{1}=4\lambda^{2}M_{2}+2{\left[\lambda \sqrt{2M_{1}}+\delta^{-1}\left(2M_{2}+1\right)+\delta^{-1}4M_{2}\right]}^{2}>0.$$

An integration over (0,t) yields

$$\int_{0}^{t}∥q_{\tau }{\left(\tau \right)∥}^{2}d\tau +{∥q_{x}\left(t\right)∥}^{2}\leq K\;t\;e^{t}\underset{\left[0,h\right]}{\sup}\left(∥{\bar{z}}_{x}{\left(t\right)∥}^{2}+{∥\bar{q}\left(t\right)∥}_{H^{1}\left(\Omega \right)}^{2}\right).$$

Finally, we multiply Equation (33) by q to obtain

$$\begin{array}{cc} \frac{1}{2}\frac{d}{dt}{∥q∥}^{2}+{∥q_{x}∥}^{2}= & -\lambda \int_{\Omega }L\left(\bar{m}\right)\cdot q\;{\bar{z}}_{x}\;dx+\lambda \int_{\Omega }L\left(-\bar{q}\right)\cdot q\;{\bar{\bar{u}}}_{x}\;dx\\ & -\delta^{-1}\int_{\Omega }\left(\right|\bar{m}{|}^{2}-1)\bar{q}\cdot q\;dx-\delta^{-1}\int_{\Omega }\bar{q}\cdot \left(\bar{\bar{m}}+\bar{m}\right)\left[\;\bar{\bar{m}}\cdot q\;\right]dx. \end{array}$$

As above, the right-hand side is estimated by replacing $q_{t}$ with q. Hence,

$$\frac{1}{2}\frac{d}{dt}{∥q\left(t\right)∥}^{2}+∥q_{x}{\left(t\right)∥}^{2}\leq K_{2}\underset{\left[0,h\right]}{\sup}\left(∥{\bar{z}}_{x}{\left(t\right)∥}^{2}+{∥\bar{q}\left(t\right)∥}_{H^{1}\left(\Omega \right)}^{2}\right)+\frac{1}{2}{∥q\left(t\right)∥}^{2}\;,$$

where

$$K_{2}=2\lambda^{2}M_{2}+{\left[\lambda \sqrt{2M_{1}}+\delta^{-1}\left(2M_{2}+1\right)+\delta^{-1}4M_{2}\right]}^{2}>0.$$

As a consequence,

$$\frac{d}{dt}{(∥q\left(t\right)∥}^{2}e^{-t})\leq K_{2}\underset{\left[0,h\right]}{\sup}\left(∥{\bar{z}}_{x}{\left(t\right)∥}^{2}+{∥\bar{q}\left(t\right)∥}_{H^{1}\left(\Omega \right)}^{2}\right),$$

which after an integration leads to

$${∥q\left(t\right)∥}^{2}\leq K_{2}te^{t}\underset{\left[0,h\right]}{\sup}\left(∥{\bar{z}}_{x}{\left(t\right)∥}^{2}+{∥\bar{q}\left(t\right)∥}_{H^{1}\left(\Omega \right)}^{2}\right)\;.$$

Collecting all the previous inequalities, the estimate (iii) follows.☐

**Theorem** **1.** *Let T>0. Given $\delta <{\left(g_{0}/\lambda \right)}^{2}$, there exists a unique solution (u,m) to the problem* (12)–(15)*, subject to conditions* (7)–(9)*, which satisfies the following conditions:*

- *$u\in C^{0}\left(\left[0,T\right];\;H_{0}^{1}\left(\Omega \right)\right)\;\cap \;C^{1}\left(\left[0,T\right];L^{2}\left(\Omega \right)\right)$;*
- *$m\in C^{0}\left(\left[0,T\right];H^{1}\left(\Omega \right)\right)\cap L^{2}\left(0,T;H^{2}\left(\Omega \right)\right)$;*
- *$m_{t}\in L^{2}\left(Q\right)$.*

**Proof.**  By virtue of the uniform estimate (27) proved in Lemma 3, it turns out that we can extended the local solution up to the given fixed time *T*. Specifically, the solution can be extended, step by step, on a sequence of time intervals $\left(t_{n},t_{n+1}\right]$ such that $t_{n+1}-t_{n}=t^{*}$; hence, the result is achieved in the limit $n\to T/t^{*}$. ☐

## 5. Conclusions

The non-linear one-dimensional magneto-viscoelasticity problem (12)–(15) is studied. It is proved to admit a unique weak solution by means of suitable estimates based on the physical involved phenomena. Thus, in particular, the established estimates are for the viscoelastic behavior of the material as well as on the magnetic field and the interaction between the two different effects. Since the model considered here also takes into account the aging of the material, this result can be applied to a wide variety of real materials, particularly when aging deteriorates some characteristic behaviors. This is the case of viscoelastic materials that undergo a transition from long to short memory. However, we considered magneto-viscoelastic solids subject to aging at constant temperature. This hypothesis narrows the field of applicability of the results, as some aging phenomena are closely connected with the thermal history of the material. A possible further development consists in taking into account also the thermal variations of the body.

The results obtained in this study represent the needed theoretical background to possible future investigations and numerical simulations. Indeed, when an existence and uniqueness result of the solution is established, then the possibility to successfully perform a numerical test is open.

## Data Availability

Not applicable.
